# BOSS: context-enhanced search for biomedical objects

**DOI:** 10.1186/1472-6947-12-S1-S7

**Published:** 2012-04-30

**Authors:** Jaehoon Choi, Donghyeon Kim, Seongsoon Kim, Sunwon Lee, Kyubum Lee, Jaewoo Kang

**Affiliations:** 1 Department of Computer Science, Korea University, Seoul, Korea

## Abstract

**Background:**

There exist many academic search solutions and most of them can be put on either ends of spectrum: general-purpose search and domain-specific "deep" search systems. The general-purpose search systems, such as PubMed, offer flexible query interface, but churn out a list of matching documents that users have to go through the results in order to find the answers to their queries. On the other hand, the "deep" search systems, such as PPI Finder and iHOP, return the precompiled results in a structured way. Their results, however, are often found only within some predefined contexts. In order to alleviate these problems, we introduce a new search engine, BOSS, Biomedical Object Search System.

**Methods:**

Unlike the conventional search systems, BOSS indexes segments, rather than documents. A segment refers to a Maximal Coherent Semantic Unit (MCSU) such as phrase, clause or sentence that is semantically coherent in the given context (e.g., biomedical objects or their relations). For a user query, BOSS finds all matching segments, identifies the objects appearing in those segments, and aggregates the segments for each object. Finally, it returns the ranked list of the objects along with their matching segments.

**Results:**

The working prototype of BOSS is available at http://boss.korea.ac.kr. The current version of BOSS has indexed abstracts of more than 20 million articles published during last 16 years from 1996 to 2011 across all science disciplines.

**Conclusion:**

BOSS fills the gap between either ends of the spectrum by allowing users to pose context-free queries and by returning a structured set of results. Furthermore, BOSS exhibits the characteristic of good scalability, just as with conventional document search engines, because it is designed to use a standard document-indexing model with minimal modifications. Considering the features, BOSS notches up the technological level of traditional solutions for search on biomedical information.

## Background

The Human Genome Project, completed in 2003, transformed the nature of biology into that of an interdisciplinary science. The project proffered a new window of opportunity for experts in other domains as well, such as computer science, statistics, and chemistry, just to name a few. Naturally, there has been a rapid increase of biomedical publications, in volume and number, in non-traditional venues, such as computer science conference proceedings, as well as in the traditional ones like core biology journals.

Approximately 1.2 million studies covering all disciplines are published each year. Among them, biomedical studies constitute about 30-35% [1]. With publications exploding in number, researchers and practitioners are now facing a new challenge. Pinpointing relevant information has become an extremely labor-intensive and time-consuming process. To address this problem, researchers have introduced search services especially concerning academic literature. Google Scholar [2] and Microsoft Academic Search [3] are well known examples. These are general-purpose academic search engines covering all topics. PubMed [4] is another well known example tailored for biomedical disciplines. Although these search engines serve as a good entry point for researchers, they produce relevant article lists only, leaving most of the information-processing task to users. For example, if one wishes to find biomedical objects that inhibit EGFR (Epidermal Growth Factor Receptor), he/she might query the search systems with "EGFR inhibitors." The systems will return thousands of articles containing the keywords EGFR and inhibitors. It is the user's job to read through the articles and manually compile the answer to the query.

On the other end of spectrum, there exist special-purpose "deep" search systems. For example, EDGAR [5] is used to extract relations between drugs and genes, PIE [6] and PPI Finder [7] are used to observe protein-protein interactions, while STRING [8] and iHOP [9] are used to find out a network of proteins. These systems extract the target relations from the articles by means of natural language processing and text-mining techniques; pre-collect and store relevant information into a database. In the query time, they produce the matching pre-compiled hit results. Although they provide more refined results than the general-purpose search engines, they have some drawbacks. First, they can only serve queries that match their objectives. For example, EDGAR maintains information related only to cancer. Similarly, PPI Finder is limited to the information on protein-protein interactions. Therefore, they are unable to serve other types of queries, such as disease-protein relations or relations among SNPs. Second, their query interface is limited in functionality. For example, iHOP accepts queries based on protein and gene names, and returns compiled results on that protein or gene. However, if a user wishes to find proteins that have a certain relation with the query protein, to express the query itself poses a problem. For example, let us further suppose that a user wants to find the proteins that 'inhibit' EGFR. The user may expresses the query as "EGFR inhibitors." However, iHOP fails to return any answer to this query, because it recognizes only a precompiled list of query terms.

**Figure 1:**
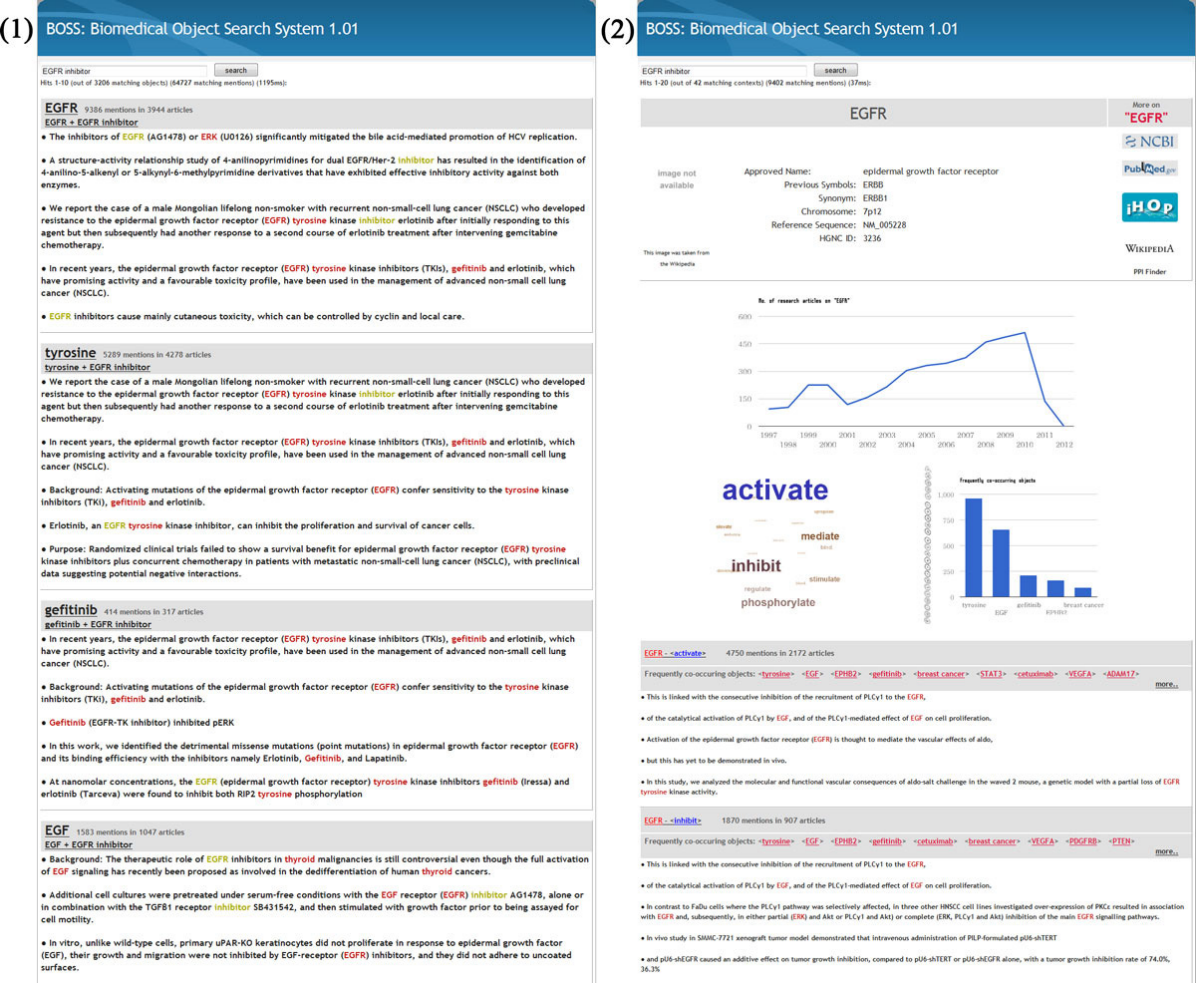
**BOSS result pages for query "EGFR inhibitors"**. (1) The main result page presenting the ranked list of objects that match to the query (2) The detail page for object EGFR.

In order to address these problems, we introduce a new paradigm for searching biomedical information. The search engine we propose, BOSS, a Biomedical Object Search System, enables free-text keyword queries just like general-purpose search engines, and produces a ranked list of relevant biomedical objects. Moreover, BOSS does not confine the results to predefined target relations (e.g., drug-gene, protein-protein, etc.). It effectively incorporates therein the benefits with the two ends of the spectrum: general-purpose and deep search systems. Figure 1 shows an example result page produced by BOSS to the query "EGFR inhibitors."

BOSS has been implemented on top of a conventional "document-retrieving" search engine with slight modifications. The major technical difference is that the indexing unit is not document, but 'Maximal Coherent Semantic Unit (MCSU)' that is a maximal subsequence of words within a document containing one coherent semantic. Hereinafter, we refer to MCSU(s) as segment(s). A single segment can be a phrase, clause, or sentence, which contains the information of an object and/or its relation to others. Once a user query is rendered, BOSS finds matching segments and classifies the results for each object and relation in the segments. Yet another benefit of this design is that we are able to achieve high scalability just as a conventional web search engine does, since we have employed the conventional inverted indexing architecture.

## Methods

**Figure 2:**
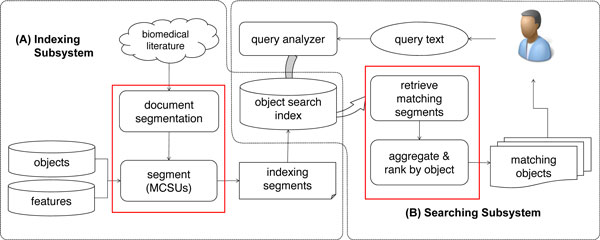
**System workflow**. BOSS has been built on top of a conventional search engine architecture with minor modification. The modified components are highlighted in red boxes.

BOSS has been implemented on top of an open source search engine, Apache Lucene 3.1.0. Lucene consists of two main subsystems: indexing and searching. BOSS implementation required modification of both the indexing model and search routines. Figure 2 illustrates the modified workflow of both subsystems. The modified components are highlighted in red boxes.

### Indexing subsystem

Like conventional search engines, BOSS indexes documents which are, in this context, the abstracts of research articles. However, unlike conventional search engines, BOSS returns matching objects, not documents. In the current version of BOSS, we manually compiled the list of objects from various biomedical information sources such as HGNC [10], GO (Gene Ontology) [11], MeSH [12], and FDA [13]. The objects include gene/protein names, symbols, synonyms, reference sequences, diseases, drugs, etc. Although we plan to implement a semi-automated object acquisition module using named entity resolution technique in the future, the present manual compilation serves the purpose well, since it is just a one-time process and furthermore we could achieve high precision through manual tuning.

We also defined the contexts of the relations among the objects, which we refer to as features. Following example explains the nature of features:

#### Example 1 *"MS-1020 **potently **inhibited **persistently-active **STAT3."*

This sentence contains two objects, MS-1020 and STAT3, and the relation between them (i.e., inhibition). The features (namely, relation words) were compiled, as follows: 1) we extract the verbs from the corpus, 2) compute the frequencies of all verbs, 3) and choose top-k frequent words and manually screen the list to eliminate inappropriate words. We used abstracts of 330,000 papers published during the first quarter of 2010 as a seed set. The feature set finally selected through this process includes 27 relation words. We term this feature set as *frequency-based feature set*.

In the initial working prototype of BOSS, we solely used the frequency-based feature set. However, we soon observed that some important but low-frequency relation words were not captured. In order to address this problem, we augmented the frequency-based feature set with a popular relation-type ontology, BioInfer [14]. Table 1 shows the final 51 features used in the current version of BOSS.

**Table 1:** The feature set used in BOSS

relation-type	NEGATIVE	FULL-STOP	BREAK-DOWN	DECREASE	INCREASE	START
features	*downregulate* inhibit suppress **repress** **interfere**	*inactivate* *halt* **block** **limit** **restrict** **kill**	*unbind* *depolymerize* *disrupt* *cleave* *disassemble*	decrease diminish **reduce**	*increase* **enrich**	*initiate * activate **promote**

relation-type	ADDITION	ASSEMBLY	UNSPECIFIED		POSITIVE	

features	*acetylate* *add* *phosphorylate*	*assemble * *cross-link * *attach * *polymerize * *bind * **integrate**	*modulate * control *regulate* interact **disseminate**	**inherit** **modify** **stabilize** **isolate**	*catalyze* *upregulate* stimulate *mediate* **accelerate**	**amplify** **elevate** **enhance** **enlarge**

The feature set is constructed by augmenting the frequency-based feature set with a popular relation-type ontology, BioInfer. The underlined features represent ones existing in both the frequency-based set and BioInfer. The bold features represent ones existing in the frequency-based set but not in BioInfer. Lastly, the italic features are ones existing in BioInfer but not in the frequency-based set.

Once the lists of objects and features are compiled, indexing is to be commenced. The first step in this regard is to segmentize the document into MCSUs. We explain the segmentation process through the example below.

#### Example 2 *"**Oral **corticosteroids decrease CC chemokine **but increase **IL-8."*

The above example contains two contradicting semantics. The first segment describes the relation between oral corticosteroids and CC chemokine, and the second segment describes their relation with IL-8. These two relations are contradicting, because the first describes "decrease" while the second points opposite. In order to produce correct answers to queries, the sentence is divided into two segments representing each maximal coherent semantic unit. For example, with the semantic segmentation, queries such as proteins that "oral corticosteroids increase" could be correctly processed to turn up IL-8, but not CC chemokine. This is the major deviation from the conventional search engines. The conventional ones index entire documents and therefore, they are incapable of producing correct results. For example, for a query, proteins that "decrease IL-8," the conventional search engines would return the document in Example 2, because the two keywords match the document. However, BOSS would return neither of the segments, as the two keywords do not match either of the segments.

In order to extract the MCSUs, we first need to split the documents into sentences. For this, we used a maximum entropy-based sentence detector in OpenNLP 1.5 package. For extracting MCSUs from the sentences, we started with a statistical parser to analyze the sentence structures. However, it turned out that statistical parsing is not scalable enough to cope with entire corpus. For this reason, in the current version of BOSS, we decided to resort to a simple heuristic rule-based algorithm as outlined in Algorithm 1.

**Algorithm 1 **MCSU Extraction Algorithm

   **procedure **MCSU_EXTRACT(sentence)

      annotate the sentence with POS tagging

      analyze the sentence structure using rule-based parser

      P = C = *ϕ*

      **if **clause exists in sentence **then**

         split sentence to clauses

         C = clauses

      **else**

         C = sentence

      **end if**

      **for all **clause in C **do**

         **if **more than one feature exist in clause **then**

            split the clause to phrases based on features

            P = P ∪ phrases

         **else**

            P = P ∪ clause

         **end if**

      **end for**

      **return **P

   **end procedure**

BOSS treats MCSUs as documents in conventional search systems and indexes them as usual. An MCSU posting list, the data structure used for inverted indexing, is formally defined as follows:

#### Definition 1 *(MCSU posting list)*

*Given an article set D *= {*d*_1_, *d*_2_, . . ., *d_n_*}, *an MCSU segment set S *= {*s*_1_, *s*_2_, . . ., *s_m_*}, *a feature set F *= {*f*_1_, *f*_2_, . . ., *f_p_*}, *and an object set O *= {*o*_1_, *o*_2_, . . ., *o_q_*}, *an MCSU posting list consists of*

$$t_{i}\Rightarrow \left[<sid_{j},\;bid_{1},\left[oid_{2},\ldots \right],\left[fid_{1},\ldots \right]>\ldots \right]$$

*where sid_j _wis the ID of MCSU segment s_j _that contains term t_i_, oid_k _is the ID of object o_k _in s_j_, fid_l _is the ID of feature f_l _in s_j_*.

Note that the structure of the index is same as that of the conventional search engines except that segments are indexed instead of documents, and the extra information of objects and features are also stored along with the segments. Only the segments that have one or more objects are indexed while features are optional. The extra information about the objects and features are used for organizing the results and ranking the entries. Moreover, we are able to retain scalability as good as conventional search engines as we have implemented our segment-based indexing using the standard inverted index architecture. We now turn to explain how BOSS computes the search results.

### Searching subsystem

BOSS returns mainly two types of result pages. The first page contains the ranked list of matching objects, along with the snippets of segments for each object. Figure 1 depicts an example page, which shows the matching objects for the query "EGFR inhibitors." An example of the second type of page is shown in Figure 1, 2. It presents detailed information of an object. The second page is shown when a user clicks on the object on the first page.

In order to compute the matching object list in the first page, we mostly follow the conventional IR system's inverted index-probing process. The main difference is that we need to aggregate the matching segment(s) for each object, and rank the objects to produce the final result. More specifically, we take the following four steps: 1) Find the matching segments by probing the index, 2) compute the scores for each segment, 3) aggregate the segments based on the objects they contain, 4) and finally compute the scores of the objects by combining the segment scores.

Scoring is one of the most important components in search. It plays a key role in enhancing the quality of the results. However, in this work, we focus on presenting the overall system architecture, and leave evaluation of the scoring function unaddressed for the future work. Instead, we used simple scoring functions in this work. The following defines the score of a segment matching to a query.

#### Definition 2 *(Segment Score)*

*Given the sets D, S, F, O in *Definition 1, *the score of segment s_j _given query q is defined as follows:*

(1) $$score_{s}\left(q,\;s_{j}\right)=coord\left(q,\;s_{j}\right)\times \underset{f_{k}\in s_{j}}{\sum }w\left(f_{k}\right)\times \underset{o_{l}\in s_{j}}{\sum }w\left(o_{l}\right)\times rc$$

*where coord*(*q*, *s_j_*) *is the coordination factor computed as the number of query terms that s_j _contains divided by the total number of terms in q; w*(*f_k_*) *is the weight of feature f_k _in s_j_; w*(*o_l_*) *is the weight of object o_l _in s_j_; rc is the recency factor based on the difference between the query time and publication date of the article d_o _containing s_j_*.

In order to approximate the amount of information in each segment, we simply factored the numbers of objects and features into the score. The recency refers to the freshness of the information. The information announced recently might be more interesting to the users than the information that is several years old and contains well known facts. In the future, we plan to explore various other options, such as information reliability. It is measured by the number of citations to the article or by the impact factor of the journal that publishes the article.

Given a query *q*, scores of all matching segments are computed using *score_s_*(*q*,.) and aggregated according to the matching objects. The object score is defined as below:

#### Definition 3 *(Object Score)*

*Given the sets S, O in *Definition 1, *and $S_{o_{j}}=\;\{s_{k}|s_{k}\in S$ that matches to query q and that contains object o_j_*}*, the score of object o_j _given query q is defined as follows:*

(2) $$score_{o}\left(q,o_{j}\right)=\underset{s_{k}\in S_{o_{j}}}{\sum }score_{s}\left(q,s_{k}\right)$$

*where score_s _*(*q*, *s_k_*) *is the segment score of s_k _given query q*.

## Results

### The working prototype

The working prototype of BOSS is available at http://boss.korea.ac.kr. The current version of BOSS uses Scopus data set for indexing. This data set consists of abstracts, metadata and citation information on peer-reviewed articles. We indexed more than 20 million articles published during last 16 years from 1996 to 2011. The articles are grouped into 28 different research categories. Among them, 4 categories are related to biomedical domains including "Medicine," "Biochemistry, Genetics and Molecular Biology," "Pharmacology, Toxicology and Pharmaceutics," and "Immunology and Microbiology." The number of journals and conference proceedings included in the 4 categories amounts almost to 14,000. Articles in these categories constitute about 30-35% of all the articles in the dataset. Nonetheless, we indexed entire dataset including all 20 million articles in all 28 categories for broader coverage.

BOSS currently runs on a cluster of 5 servers, each of which consists of a dual-core 2.00 GHz CPUs, 4 GB RAM, and 1TB of disks. The total size of the index at the time of writing is close to 3.7 GB. We constantly add more articles into the index as new ones become available.

### A use case

BOSS can serve as an effective interface to a large academic corpus. It can be used as a stand-alone query service as well as a complementary tool for existing ones. For example, a user is interested in 'acute myeloid leukemia (AML).' The user can acquire relevant information effectively through curated databases, such as KEGG pathway [15] and NCBI OMIM [16]. However, due to the nature of manual curation, the curated sources may include only directly relevant information and are likely to lack remotely related information and/or relatively new discoveries.

**Figure 3:**
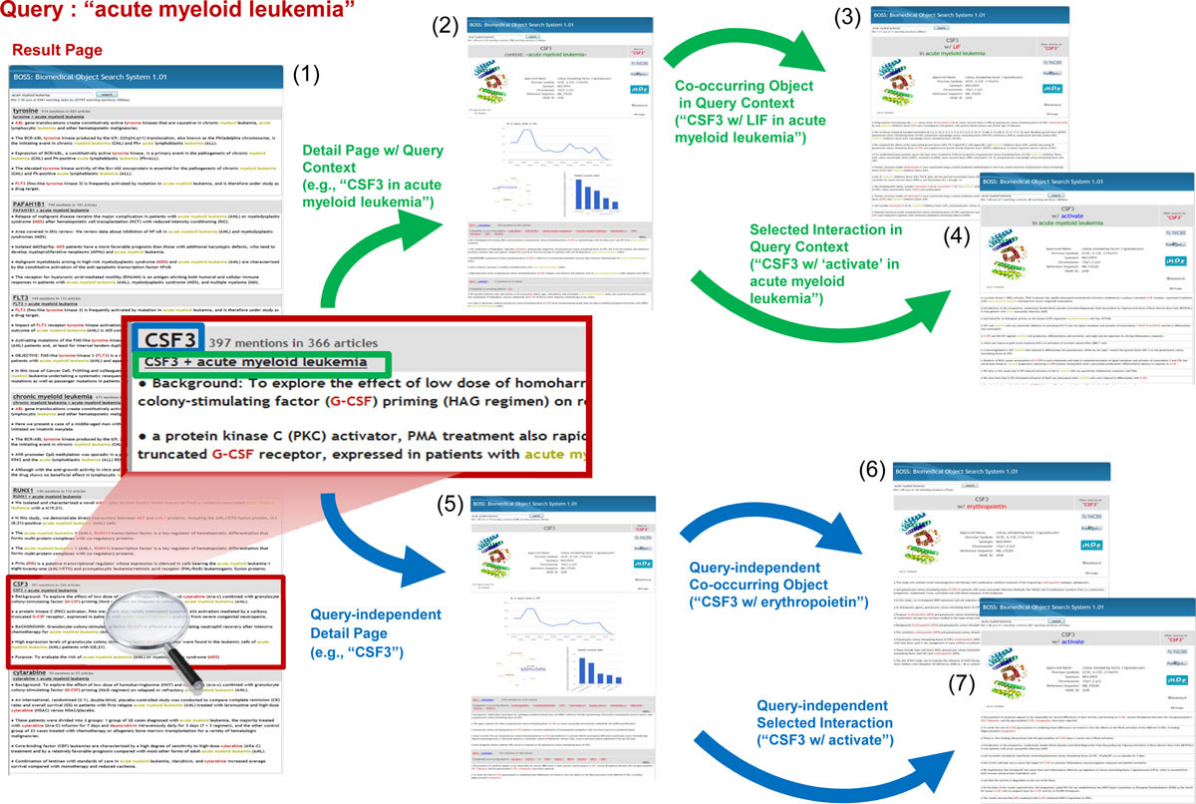
**A use case with query "acute myeloid leukemia"**. (1) The main result page (2) Detail page in query context (3) Detail page w/co-occurring object in query context (4) Detail page w/selected interaction in query context (5) Query-independent detail page (6) Query-independent detail page w/co-occurring object (7) Query-independent detail page w/selected interaction.

BOSS can complement these by its broader coverage and timely updates. In fact, BOSS has produced a number of AML-related genes that were not listed in either KEGG pathway or OMIM. For example, the 6*^th ^*ranked object for query 'acute myeloid leukemia,' CSF3 (Colony Stimulating Factor 3), was not found in either of the two curated sources (Figure 3-(1)). CSF3 is reported to affect AML patients' neutrophil recovery after chemotherapy [17]. As shown in Figure 3-(1), each entry in the result page consists of two links to the corresponding object (protein, drug, etc.) and five snippets extracted from supporting articles. The expression next to the first link, "397 mentions in 366 articles," means that among all segments matching to the query, 397 segments in 366 articles mentioned CSF3.

If the user wants to learn more about the object, the user can click on the first link, "CSF3," which leads to a detail page presenting CSF3-related information (Figure 3-(5)). The second link, "CSF3 + acute myeloid leukemia," leads to the same CSF3 detail page except that the page presents CSF3-related information within the query context, 'acute myeloid leukemia' (Figure 3-(2)). The detail pages contain statistics such as the number of articles discussing CSF3 per year (line chart), interactions mentioned with CSF3 (term cloud), and frequently co-occurring objects with CSF3 (bar chart).

The user can click on the bar chart or the co-occurring object list to see the object within the context of the co-occurring object. Figure 3-(3) shows the example page of CSF3 in the context of co-occurring object LIF and the query 'acute myeloid leukemia.' LIF, leukemia inhibitory factor, whose relation with CSF3 is quite obvious in the query context, was the second most frequently co-occurring object. The most frequent co-occurring object was cytarabine, a chemotherapy agent used in the treatment of AML and non-hodgkin lymphoma [18] (bar chart in Figure 3-(2)). It seems natural that cytarabine co-occurs frequently with CSF3 that affects AML patients' neutrophil recovery.

On the other hand, Figure 3-(5) illustrates the query-independent detail page of CSF3. In this case, the most frequently co-occurring object was erythropoietin that is a glycoprotein hormone which controls red blood cell production. Figure 3-(6) shows the detail page of CSF3 in the context of erythropoietin independent of the query context. Finally, in order to see the object in the context of interaction, the user can click on the interaction link, for example "CSF3 - *<*activate *>*." Figure 3-(4) shows the page presenting CSF3 with 'activate' in the context of acute myeloid leukemia while Figure 3-(7) shows the same independent of the query context.

## Discussion

For general-purpose search on academic literature, there exist open-domain search engines such as Google Scholar and Microsoft Academic Search, and domain-specific search engines such as PubMed. There also exist many special-purpose deep search systems that provide information pre-extracted from academic references, such as PPI-finder [7] for protein-protein interactions, and STRING [8] and iHOP [9] for protein networks.

Apart from these efforts, there also have been attempts to address the "object search" problem mainly by database communities. Chakrabarti et al. proposed a method for mapping keywords to objects indirectly through the documents that describe the target objects [19]. Cheng et al. employed the information extraction technique to locate object instances from documents for each predefined target entity type, and returns the matching object instances for queries in the form of *<*keyword, entity type *>*(e.g., "Amazon #phone" for retrieving phone numbers of Amazon.com) [20,21]. The relevance scores for the matching instances are computed based on the frequencies and proximity to the matching keywords.

The major difference between our work and these object search systems lies in the explicit handling of relations. The previous object search systems tackle the implicitly defined "ISA" relation alone. For example, what returned to the query "Amazon #phone" are the phone numbers of Amazon. In the field of biomedical application, it is important to understand the relational context for each object instance. However, considering the structure of the previous systems, it would be very difficult to support queries that contain explicit relations, such as biomedical objects that "inhibit" EGFR. In order to support those queries, the relation between the keyword and the matching objects should be explicitly defined. Moreover, the context of the relations should be defined not by proximity, but by semantics. For example, let us suppose that we have a sentence "the protein A promotes the protein B, while suppresses the protein C." For the query, proteins that "the protein A suppresses" return the protein C alone without the protein B. The inability under the previous solutions comes from the failure to define relations explicitly and to define semantics-driven context for the relations.

## Conclusion

We introduced a new platform, BOSS, for search on biomedical objects. BOSS is designed to fill the gap between the two opposite ends of the spectrum: general-purpose and domain-specific deep search systems. The general-purpose systems, such as PubMed, allow users to express any types of keyword queries; however, they simply return numerous matching documents, and leave the rest of the query-answering task to users. On the other hand, the deep search systems, such as iHOP and PPI Finder, produce precompiled information in a structured way; however, the precompiled information is typically limited to some predefined contexts, and, thus, they are incapable of answering queries outside of the confinement.

BOSS enables users to freely express any types of queries, and still returns the matching results in a structured way. Furthermore, since it is implemented on top of a conventional information retrieval system upon straightforward extension of its indexing model, BOSS achieves scalability as high as the conventional document search engines offer. In the future, we plan to investigate scoring methods for ranking objects in order to further refine the quality of the results.

## List of abbreviations used

BOSS: Biomedical Object Search System; MCSU: Maximal Coherent Semantic Unit; EGFR: Epidermal Growth Factor Receptor; AML: Acute Myeloid Leukemia; CSF3: Colony Stimulating Factor 3; LIF: Leukemia Inhibitory Factor.

## Competing interests

The authors declare that they have no competing interests.

## Authors' contributions

JC and JK carried out the design of the system and drafted the manuscript. DK and SK participated in the implementation of the system and its validation. SL and KL carried out the use of the system for validation and helped to draft the manuscript. All authors read and approved the final manuscript.
